# Dexmedetomidine alleviates sleep-restriction-mediated exaggeration of postoperative immunosuppression via splenic TFF2 in aged mice

**DOI:** 10.18632/aging.102952

**Published:** 2020-03-22

**Authors:** Guangzhi Wang, Xiaoying Wu, Guosong Zhu, Shuangyin Han, Jiaqiang Zhang

**Affiliations:** 1 Department of Anesthesiology, Henan Provincial People’s Hospital, Zhengzhou University People’s Hospital, Zhengzhou 450003, Henan, China; 2 Department of Gastroenterology, Henan Provincial People’s Hospital, Zhengzhou University People’s Hospital, Zhengzhou 450003, Henan, China

**Keywords:** gut flora, macrophage, myeloid-derived suppressor cells, postoperative immunosuppression, sleep-restriction

## Abstract

Major abdominal procedures could induce dysfunction in the immune system and lead to postoperative immunosuppression. Sleep dysfunction is associated with impaired immune activity. However, the effects of postoperative sleep dysfunction on postoperative immune function remain unclear. In this study, we found that sleep-restriction (SR) after surgery increased the spleen weight and the percentage of myeloid-derived suppressor cells (MDSCs) in the spleen, and inhibited splenic CD8^+^ T cells activity, which was via inhibiting subdiaphragmatic vagus nerve (SVN)-mediated trefoil factor 2 (TFF2) expression in the spleen of aged mice. Dexmedetomidine could alleviate SR-induced these changes via modulating gut microbiota, which acted through SVN. Moreover, we showed essential roles of splenic TFF2 in attenuating SR-induced reduced protective ability against Escherichia coli (*E. coli*) pneumonia, increased expression of IL-4 and IL-13 in the lung and M2 polarization of alveolar macrophages (AMs), and decreased phagocytic activity of AMs. Dexmedetomidine improved SR-induced reduced protective ability against *E. coli* pneumonia via splenic TFF2, and subsequently decreasing IL-4 and IL-13 expression in the lung via modulating gut microbiota/SVN, increasing the compromised phagocytic activity of AMs, and ultimately decreasing M2 polarization of AMs. Taken together, dexmedetomidine-induced increase in splenic TFF2 expresssion could alleviate SR-induced exaggeration of postoperative immunosuppression.

## INTRODUCTION

Major surgery and anaesthesia with severe tissue trauma are strongly associated with extensive immunosuppression in the immediate post-operative period, which arises within a few hours of surgery and lasts for several days, and has been implicated in an increased risk of infectious complications and tumour metastasis formation [1–3]. Despite recent progress in understanding and treatment of postoperative immunosuppression, the postoperative incidence of immunosuppression after major surgery, especially in older adults, remains high and the strategies against immunosuppression are inadequate.

In the hospital, disease-related symptoms, environmental factors and health care practices, including postoperative pain, patient discomfort, noise, continuous ambient light, and frequent performance of vital sign measures and tests, contribute to sleep disorders [4]. Several studies have demonstrated that patients suffer with poor sleep quality during the first postoperative week [5–8]. Sleep is particularly important for initiating effective adaptive immune responses [9], and disrupted sleep is associated with immune dysfunction, impaired resistance to infection [10]. Sleep disruption can activate the sympathetic nervous system (SNS) and the hypothalamic-pituitary-adrenal (HPA) axis and increase evening cortisol levels [11, 12], which could inhibit innate and adaptive immune responses [13–15]. In one study including 42 medically and psychiatrically healthy volunteers, acute sleep deprivation resulted in almost a 50% decrease in natural killer cell activity and a 50% decrease in lymphokine killer cell activity [16]. In addition, it is shown that sleep deprivation (SD) severely disturbs the functional rhythm of regulatory T cells (Tregs) [17]. However, the effects and mechanisms of postoperative sleep disruption on the recovery of immunosuppression remain unclear.

It has been shown that both chronic and acute sleep disruption could induce alterations in the gut microbiota composition [18–20]. There is emerging evidence that gut microbiota not only controls intestinal immunity but extra-intestinal immunity as well [21]. Previous studies have demonstrated that gut microbes play critical roles in maintaining a steady-state granulopoiesis in immunosuppression states [22], effecting innate immunity and adaptive immune homeostasis [23–25], and influencing pulmonary host defense [26, 27]. Evidences have shown that subdiaphragmatic vagus nerve (SVN) is a well-established pathway for the communication of gut microbiota with extra-intestinal organs such as brain [28, 29].

Evidence has shown that SVN-mediated trefoil factor 2 (TFF2) secretion from memory T cells in the spleen, which could inhibit the expansion of myeloid-derived suppressor cells (MDSCs) and liberate anti-tumorigenic CD8^+^ T cells to suppress colonic carcinogenesis [30]. Splenic denervation or deletion of TFF2 results in the expansion of MDSCs and colorectal cancer [30], indicating SVN-mediated splenic TFF2 expression plays essential role in the inhibition of MDSCs proliferation and immunosuppression. However, whether such pathway is involved in the pathological mechanism of postoperative immunosuppression is unclear.

Based on above previous studies, this study aims to investigate whether postoperative sleep deprivation could exaggerate immunosuppression through gut microbiota disturbance-induced inhibition of splenic TFF2 expression and subsequent promotion of MDSCs proliferation in aged mice. Moreover, we further sought to investigate whether sleep-restriction (SR)-induced exaggeration of postoperative immunosuppression could be alleviated after treatment with dexmedetomidine, which is shown to be able to promote non-rapid eye movement (NREM) stage 3 sleep in human [31].

## RESULTS

### SR resulted in splenomegaly, increased MDSCs expansion and decreased splenic CD8^+^ cells activity via inhibiting splenic TFF2 after surgery

**Figure 1:**
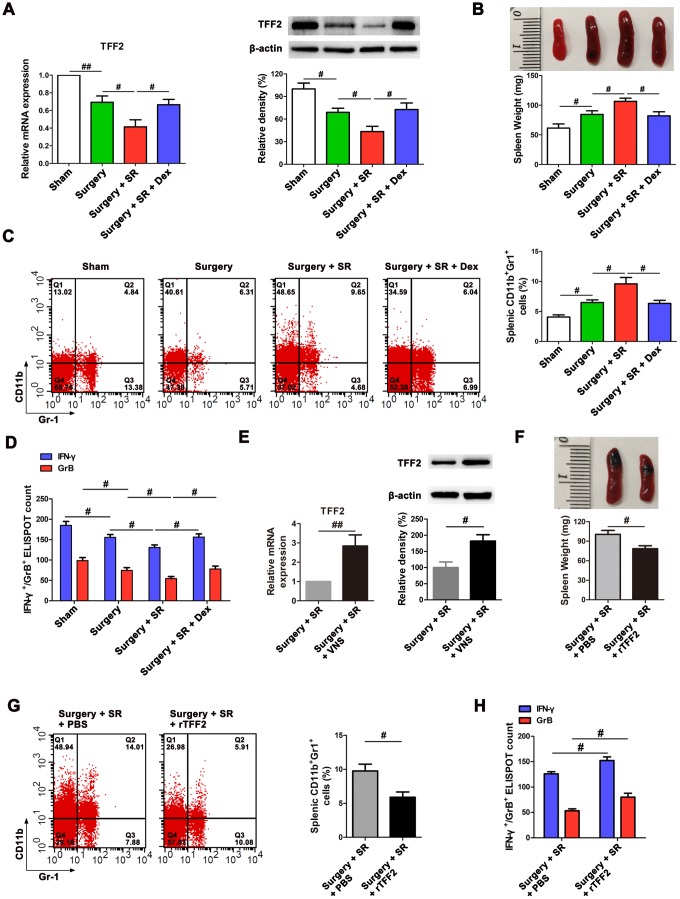
**Postoperative sleep-restriction (SR) increased myeloid-derived suppressor cells (MDSCs) expansion and decreased splenic CD8^+^ cells activity via inhibiting splenic trefoil factor 2 (TFF2).** (**A**) The expression of TFF2 in the spleen of SR mice with or without dexmedetomidine (Dex) treatment was analysed by real-time PCR (RT-PCR and western blotting. (**B**) Spleen weight in each group after 7 days of SR. (**C**) Flow cytometry analysis of spleen for CD11b^+^ Gr-1^+^ MDSCs in SR mice with or without Dex treatment. (**D**) The enzyme-linked immunospot (ELISPOT) assay of the levels of interferon-γ (IFN-γ) and Granzyme B (GrB) in splenic CD8^+^ T cells from SR mice with or without Dex treatment. (**E**) The mRNA and protein expression of TFF2 in the spleen were analysed by RT-PCR and western blotting in SR mice with or without vagus nerve stimulation (VNS). (**F**) Spleen weight in SR mice with the treatment of recombinant human TFF2 protein (rTFF2) or PBS. (**G**) Flow cytometry analysis of spleen for CD11b^+^ Gr-1^+^ MDSCs in SR mice with the treatment of rTFF2 or PBS. (**H**) ELISPOT assay of the levels of IFN-γ and GrB in splenic CD8^+^ T cells from SR mice with the treatment of rTFF2 or PBS. All data represent mean ± SEM, n = 5; ^#^*P* < 0.05, ^##^*P* < 0.01.

Splenic TFF2 plays important roles in inhibiting MDSCs expansion and CD8^+^ T cells activity in the spleen, which is strongly associated with systemic immune function [30]. We therefore first assessed the roles of postoperative SR in splenic TFF2 expression, MDSCs expansion and CD8^+^ T cells activity in aged mice. There was a significant decreased mRNA and protein expression of splenic TFF2 (*P* < 0.01 and *P* < 0.05, respectively; Figure 1A), along with increased spleen weight (*P* < 0.05; Figure 1B) and MDSCs percentage in the spleen (*P* < 0.05; Figure 1C) in surgery mice compared with that in sham-operated mice. Splenic CD8^+^ T cells from surgery mice expressed lower levels of interferon-γ (IFN-γ) and Granzyme B (GrB) compared with splenic CD8^+^ T cells from sham-operated mice (*P* < 0.05 and *P* < 0.05, respectively; Figure 1D). Postoperative SR resulted in a further decrease in mRNA and protein expression of splenic TFF2 (*P* < 0.05 and *P* < 0.05, respectively; Figure 1A), and a further increase in spleen weight (*P* < 0.05; Figure 1B) and MDSCs percentage in the spleen (*P* < 0.05; Figure 1C). Splenic CD8^+^ T cells from postoperative SR mice expressed lower levels of IFN-γ and GrB compared with splenic CD8^+^ T cells from surgery mice (*P* < 0.05 and *P* < 0.05, respectively; Figure 1D). However, dexmedetomidine treatment during SR abrogated SR-induced decrease in splenic TFF2 expression and increase in spleen weight and MDSCs percentage in the spleen (all *P* < 0.05; Figure 1A–1D).

SVN is important for splenic TFF2 expression in tumor-mediated immunosuppression [30]. We hypothesized that splenic TFF2 expression was also inhibited by the vagus nerve. Indeed, vagal nerve stimulation (VNS) increased the mRNA and protein expression of splenic TFF2 (*P* < 0.01 and *P* < 0.05, respectively; Figure 1E).

Splenic TFF2 plays key roles in inhibiting MDSCs expansion in the spleen and thus increasing CD8^+^ T cells activity in tumor-mediated immunosuppression. Consistently, we found that recombinant human TFF2 protein (rTFF2) treatment to postoperative SR mice led to a decrease in spleen weight (*P* < 0.05; Figure 1F) and MDSCs percentage in the spleen (*P* < 0.05; Figure 1G). Splenic CD8^+^ T cells from rTFF2-treated SR mice express higher levels of IFN-γ and GrB compared with splenic CD8^+^ T cells from SR mice (*P* < 0.05 and *P* < 0.05, respectively; Figure 1H).

### Dexmedetomidine attenuated SR-induced decreased splenic TFF2 expression, increased MDSCs expansion and decreased CD8^+^ T cells activity via SVN

**Figure 2:**
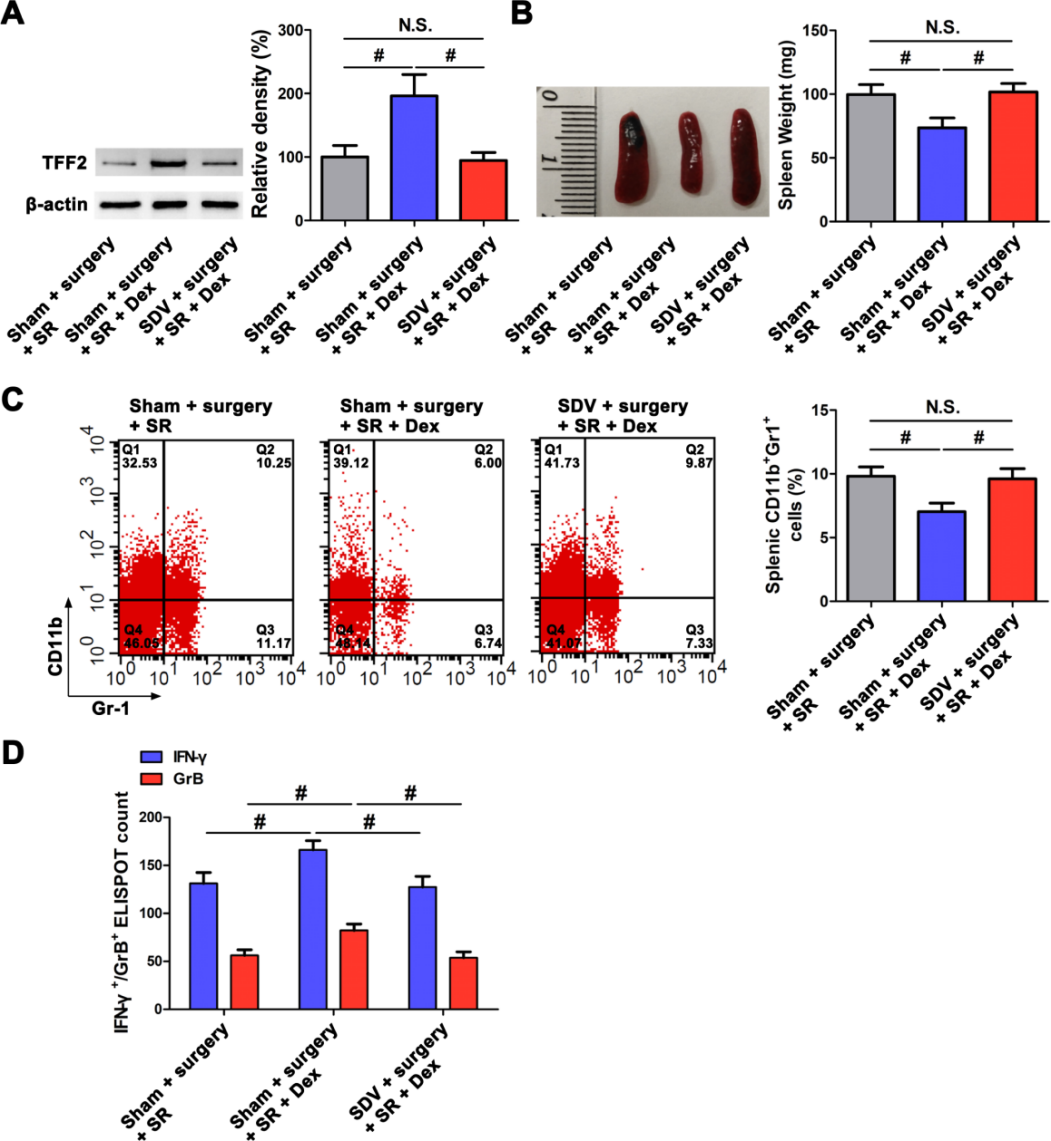
**Subdiaphragmatic vagus nerve mediated the attenuated effects of dexmedetomidine (Dex) on sleep-restriction (SR)-induced decrease in splenic trefoil factor 2 (TFF2) expression, increase in the expansion of myeloid-derived suppressor cells (MDSCs) and decrease in CD8^+^ T cells activity.** (**A**) The expression of TFF2 in the spleen of dexmedetomidine (Dex)-treated SR mice with or without bilateral sub-diaphragmatic vagotomy (SDV) was analysed by western blotting. (**B**) spleen weight in Dex-treated SR mice with or without SDV. (**C**) Flow cytometry analysis of spleen for CD11b^+^ Gr-1^+^ MDSCs in Dex-treated SR mice with or without SDV. (**D**) The enzyme-linked immunospot (ELISPOT) assay of the levels of interferon-γ (IFN-γ) and Granzyme B (GrB) in splenic CD8^+^ T cells from Dex-treated SR mice with or without SDV. All data represent mean ± SEM, n = 5; ^#^*P* < 0.05, N.S., not significant.

After sub-diaphragmatic vagotomy (SDV), dexmedetomidine-treated SR mice showed a significant decrease in splenic TFF2 mRNA and protein expression (*P* < 0.05 and *P* < 0.05, respectively; Figure 2A), an increase in the spleen weight (*P* < 0.05; Figure 2B) and a decrease in MDSCs percentage in the spleen (*P* < 0.05; Figure 2C). Splenic CD8^+^ T cells from dexmedetomidine-treated SR mice with SDV express lower levels of IFN-γ and GrB compared with splenic CD8^+^ T cells from dexmedetomidine-treated SR mice without SDV (*P* < 0.05 and *P* < 0.05, respectively; Figure 2D).

### Dexmedetomidine attenuated SR-induced expansion of MDSCs and decrease in CD8^+^ T cells activity via improving gut microbiota

**Figure 3:**
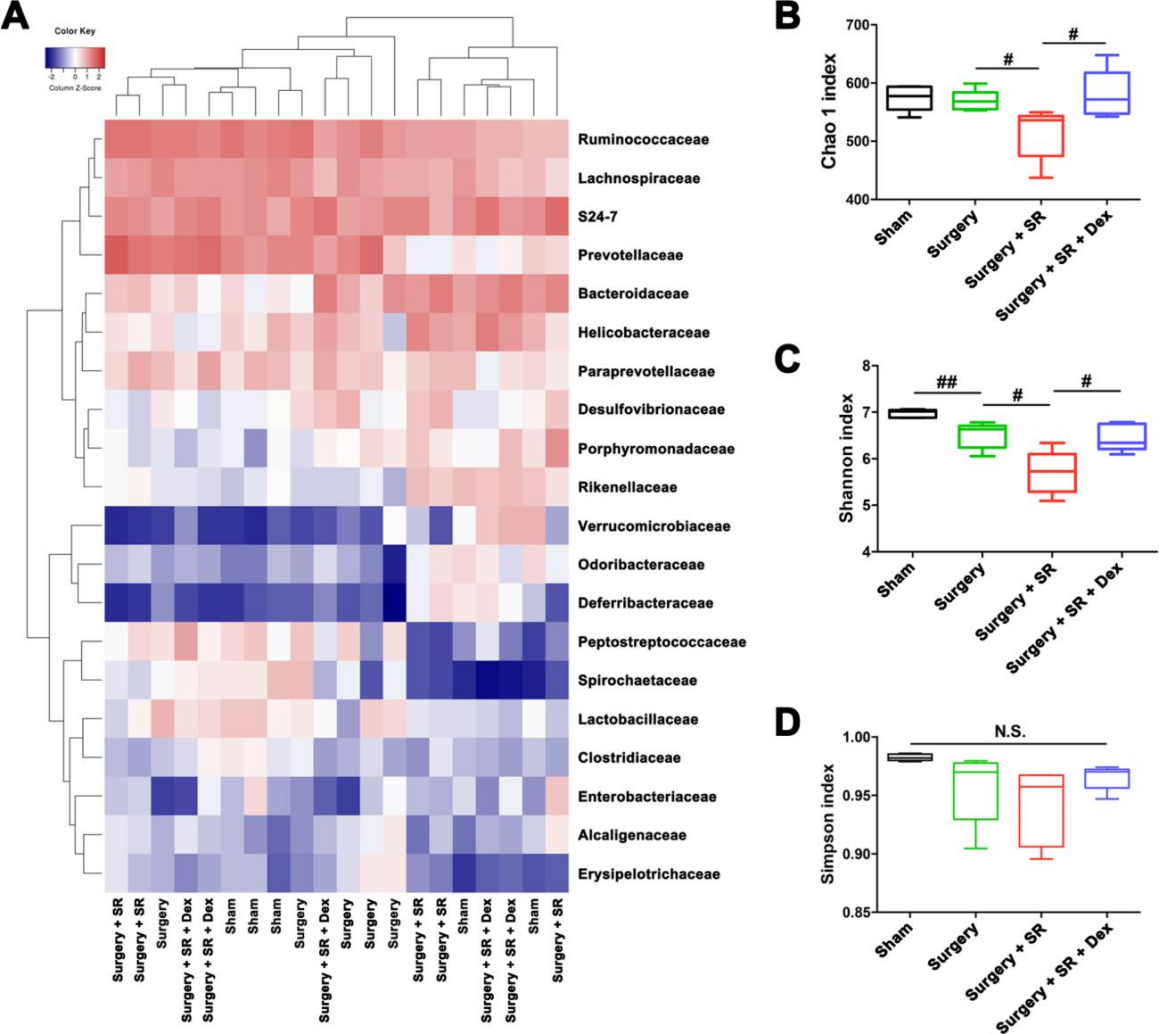
**Dexmedetomidine (Dex) treatment improved sleep-restriction (SR)-induced exaggeration of postoperative gut microbiota disturbance.** (**A**) Heat map of differential levels of bacteria between the groups. (**B**) Chao 1 index. (**C**) Shannon index. (**D**) Simpson index. Data represents mean ± SEM, n = 5; ^#^*P* < 0.05, ^##^*P* < 0.01. N.S., not significant.

Given the previously demonstrated effects of sleep disruption on gut microbiota composition [18–20] and the important roles of gut microbiota in systemic immune function [23–25], we performed 16S rRNA gene sequence analysis and fecal microbiota transplantation (FMT) to determine the effects of gut microbiota on postoperative immunosuppression. The Shannon index was significantly lower in fecal samples from sham-operated mice than that from surgery mice (*P* < 0.01; Figure 3A, 3C), although there was no significant difference in the Chao 1 index and Simpson index between the two groups (Figure 3B, 3D). Moreover, there was a significant decreased Chao 1 index (*P* < 0.05; Figure 3B) and Shannon index (*P* < 0.05; Figure 3C) in postoperative SR mice compared with surgery mice. However, dexmedetomidine-treated SR mice showed a significant increase in the Chao 1 index (*P* < 0.05; Figure 3B) and Shannon index (*P* < 0.05; Figure 3C), indicating dexmedetomidine could improve gut microbiota disturbance after postoperative SR.

**Figure 4:**
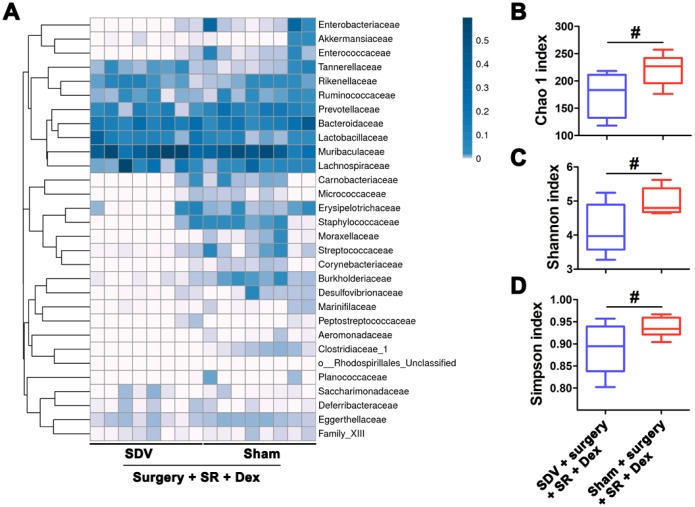
**Sub-diaphragmatic vagotomy (SDV) increased postoperative gut microbiota disturbance in dexmedetomidine (Dex)-treated sleep-restriction (SR) mice.** (**A**) Heat map of differential levels of bacteria between the groups. (**B**) Chao 1 index. (**C**) Shannon index. (**D**) Simpson index. Data represents mean ± SEM, n = 8; ^#^*P* < 0.05.

Moreover, we found that the Chao 1 index, Shannon index and Simpson index were decreased in postoperative SR mice received SDV compared with postoperative SR mice received sham surgery (all *P* < 0.05; Figure 4).

**Figure 5:**
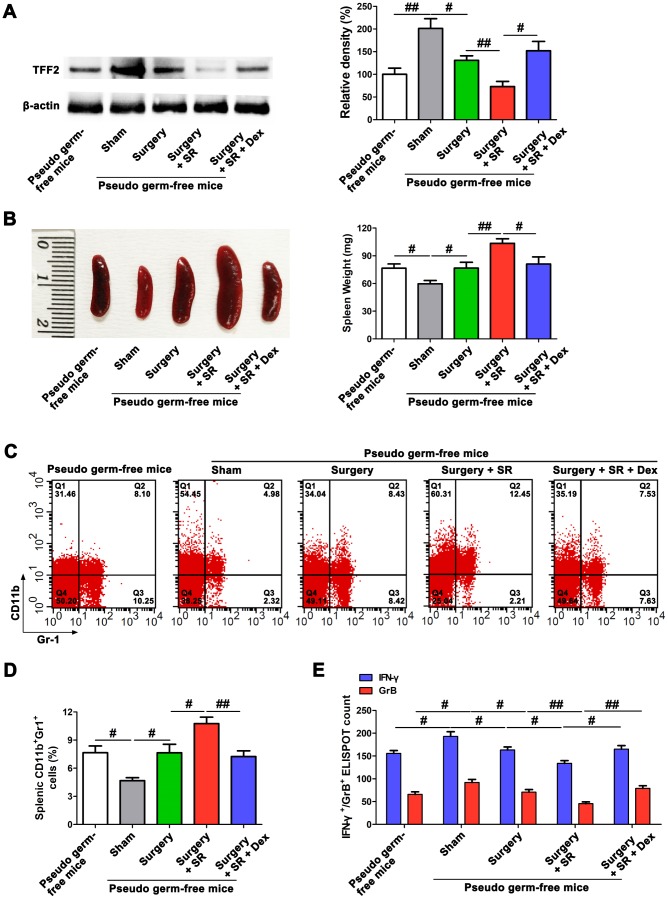
**Dexmedetomidine (Dex) attenuated postoperative sleep-restriction (SR)-induced decrease in splenic trefoil factor 2 (TFF2) expression, increase in myeloid-derived suppressor cells (MDSCs) expansion and decrease in splenic CD8^+^ cells activity via improving gut microbiota disturbance.** (**A**) Western blotting analysis of splenic TFF2 expression in sham-operated mice and pseudo-germ-free mouse received fecal microbiota transplantation (FMT). (**B**) Spleen weight in sham-operated mice and pseudo-germ-free mouse received FMT. (**C**, **D**) Flow cytometry analysis of spleen for CD11b^+^ Gr-1^+^ MDSCs in sham-operated mice and pseudo-germ-free mouse received FMT. (**E**) The enzyme-linked immunospot (ELISPOT) assay of the levels of interferon-γ (IFN-γ) and Granzyme B (GrB) in splenic CD8^+^ T cells from sham-operated mice and pseudo-germ-free mouse received FMT. All data represent mean ± SEM, n = 5; ^#^*P* < 0.05, ^##^*P* < 0.01.

To further elucidate the role of gut microbiota in the promoting effects of dexmedetomidine on splenic TFF2 expression after SR, we created a pseudo germ-free mouse model by administering antibiotics for 14 consecutive days, and pseudo germ-free mice were then received FMT for 14 consecutive days. We found that pseudo germ-free mice received FMT with feces of sham-operated mice increased the protein expression of splenic TFF2 expression (*P* < 0.01; Figure 5A), decreased spleen weight (*P* < 0.05; Figure 5B) and MDSCs percentage (*P* < 0.05; Figure 5C, 5D), compared to pseudo germ-free mice without FMT. Splenic CD8^+^ T cells from pseudo germ-free mice received FMT with feces of sham-operated mice express higher levels of IFN-γ and GrB compared with splenic CD8^+^ T cells from pseudo germ-free mice without FMT (*P* < 0.05 and *P* < 0.05, respectively; Figure 5E). Gut microbiota transplant from surgery mice to pseudo germ-free mice significantly decreased splenic TFF2 expression (*P* < 0.05; Figure 5A), increased spleen weight (*P* < 0.05; Figure 5B) and MDSCs percentage (*P* < 0.05; Figure 5C, 5D). Splenic CD8^+^ T cells from pseudo germ-free mice received FMT with feces of surgery mice express lower levels of IFN-γ and GrB compared with splenic CD8^+^ T cells from pseudo germ-free mice received FMT with feces of sham-operated mice (*P* < 0.05 and *P* < 0.05, respectively; Figure 5E). There was a significant decreased expression of splenic TFF2 (*P* < 0.01; Figure 5A, 5B), along with increased spleen weight (*P* < 0.01; Figure 5B) and MDSCs percentage in the spleen (*P* < 0.05; Figure 5C, 5D) in pseudo germ-free mice received FMT with feces of postoperative SR mice compared with that in pseudo germ-free mice received FMT with feces of surgery mice. Splenic CD8^+^ T cells from pseudo germ-free mice received FMT with feces of postoperative SR mice express lower levels of IFN-γ and GrB compared with splenic CD8^+^ T cells from pseudo germ-free mice received FMT with feces of surgery mice (*P* < 0.05 and *P* < 0.01, respectively; Figure 5E). Interestingly, pseudo germ-free mice received FMT with feces of dexmedetomidine-treated SR mice showed a significant increased expression of splenic TFF2 (*P* < 0.05; Figure 5A, 5B), along with increased spleen weight (*P* < 0.05; Figure 5B) and MDSCs percentage in the spleen (*P* < 0.01; Figure 5C, 5D) compared with pseudo germ-free mice received FMT with feces of postoperative SR mice. Splenic CD8^+^ T cells from pseudo germ-free mice received FMT with feces of dexmedetomidine-treated SR mice express higher levels of IFN-γ and GrB compared with splenic CD8^+^ T cells from pseudo germ-free mice received FMT with feces of postoperative SR mice (*P* < 0.05 and *P* < 0.01, respectively; Figure 5E).

### SVN served as a bridge between gut microbiota and spleen after postoperative SR

**Figure 6:**
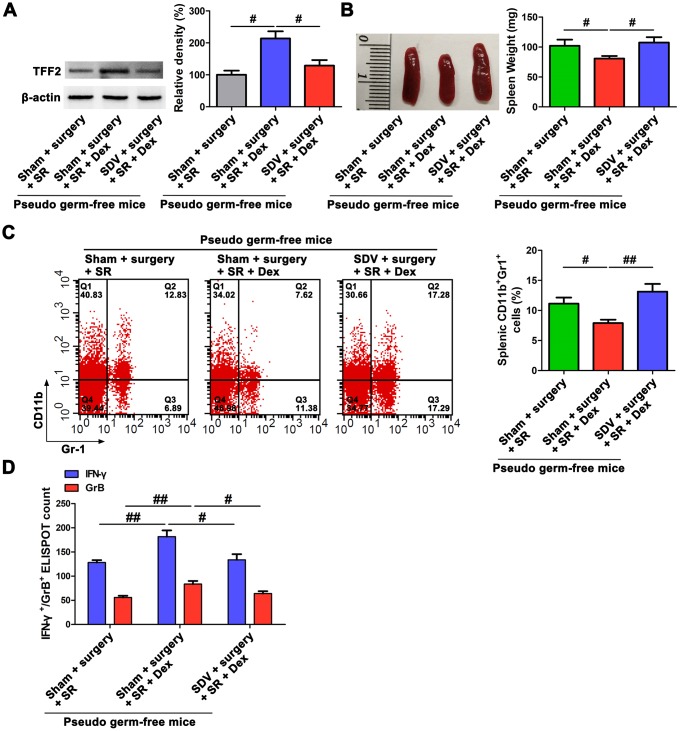
**Subdiaphragmatic vagus nerve served as a bridge between gut microbiota and spleen after postoperative sleep-restriction (SR).** (**A**) Western blotting analysis of splenic trefoil factor 2 (TFF2) expression in pseudo-germ-free mouse received fecal microbiota transplantation (FMT) with feces of SR mice, dexmedetomidine (Dex)-treated SR mice or Dex-treated SR mice with sub-diaphragmatic vagotomy (SDV). (**B**) Spleen weight in pseudo-germ-free mouse received FMT with feces of SR mice, Dex-treated SR mice or Dex-treated SR mice with SDV. (**C**) Flow cytometry analysis of spleen for CD11b^+^ Gr-1^+^ myeloid-derived suppressor cells (MDSCs) in pseudo-germ-free mouse received FMT with feces of SR mice, Dex-treated SR mice or Dex-treated SR mice with SDV. (**D**) The enzyme-linked immunospot (ELISPOT) assay of the levels of interferon-γ (IFN-γ) and Granzyme B (GrB) in splenic CD8^+^ T cells from pseudo-germ-free mouse received FMT with feces of SR mice, Dex-treated SR mice or Dex-treated SR mice with SDV. All data represent mean ± SEM, n = 5; ^#^*P* < 0.05, ^##^*P* < 0.01.

Given the findings that SVN is a well-established pathway for the communication of gut microbiota with extra-intestinal organs [28, 29], and combined with our above results that both gut microbiota and SVN were involved in the promoting effects of dexmedetomidine on splenic TFF2 expression and CD8^+^ Tcells activity after SR, next we asked whether SVN could serve as a bridge between gut microbiota and spleen in dexmedetomidine-treated mice. Interestingly, we found that SDV abrogated dexmedetomidine treatment-mediated increase in splenic TFF2 mRNA and protein expression (*P* < 0.05 and *P* < 0.05, respectively; Figure 6A), decrease in spleen weight (*P* < 0.05; Figure 6B) and MDSCs percentage in the spleen (*P* < 0.01; Figure 6C) after SR. Splenic CD8^+^ T cells from dexmedetomidine-treated mice with SDV express lower levels of IFN-γ and GrB compared with splenic CD8^+^ T cells from dexmedetomidine-treated mice without SDV (*P* < 0.05 and *P* < 0.05, respectively; Figure 6D).

### Dexmedetomidine improved postoperative SR-induced reduction of protective ability against *E. coli* pneumonia through splenic TFF2

**Figure 7:**
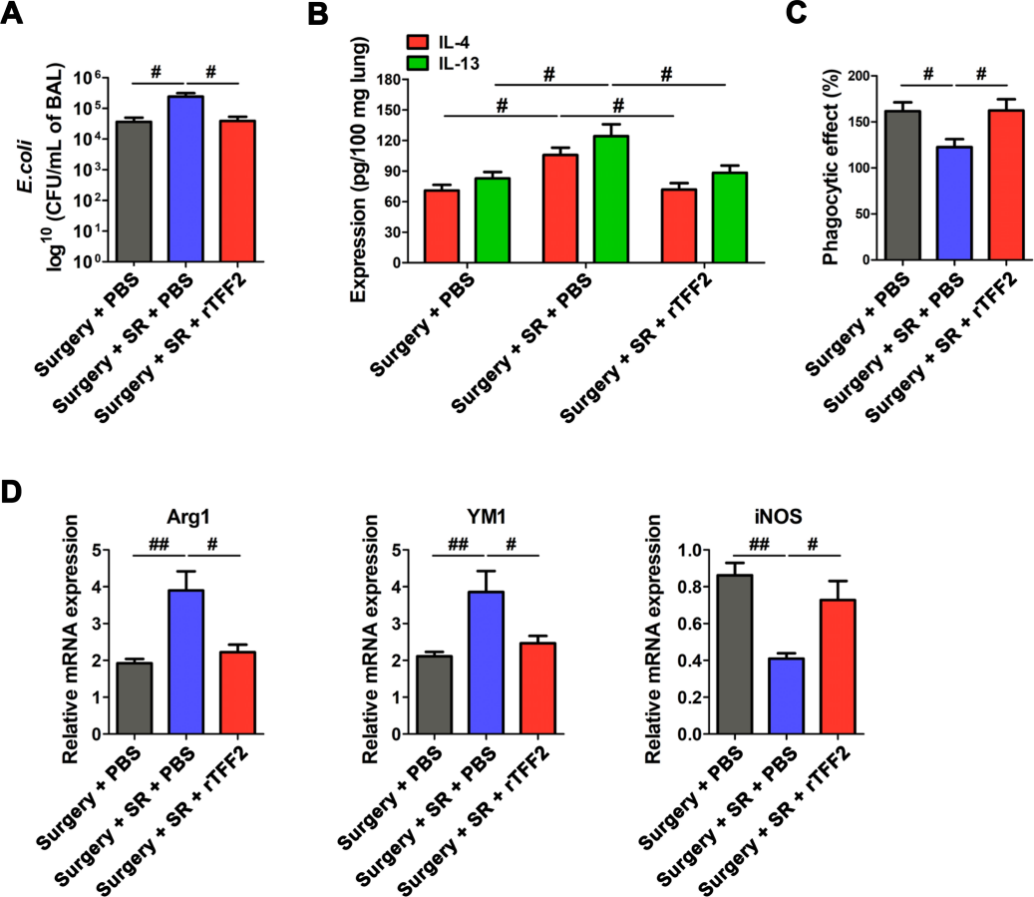
**Splenic trefoil factor 2 (TFF2) was essential to attenuate SR-induced reduced protective ability against Escherichia coli (*E. coli*) pneumonia, increased expression of IL-4 and IL-13 in the lung and M2 polarization of alveolar macrophages (AMs), and decreased phagocytic activity of AMs.** (**A**) Enumeration of colony-forming units (CFU) per milliliter of bronchoalveolar lavage analyzed one day after *E. coli* pneumonia in postoperative SR mice treated with recombinant human TFF2 protein (rTFF2) or PBS. (**B**) ELISA determination of the concentrations of IL-4 and IL-13 in the lungs of postoperative SR mice treated with rTFF2 or PBS one day after *E. coli* pneumonia. (**C**) The phagocytic activity of alveolar macrophages from postoperative SR mice treated with rTFF2 or PBS. (**D**) Real-time PCR (RT-PCR) analysis of the mRNA expression of Arg1, YM and iNOS in alveolar macrophages from postoperative SR mice treated with rTFF2 or PBS. All data represent mean ± SEM, n = 5; ^#^*P* < 0.05, ^##^*P* < 0.01.

To investigate the detrimental effects of decreased splenic TFF2 after surgery on the function of immune defenses against pathogens, we induced primary pneumonia with Escherichia coli (*E.coli*). We found that rTFF2 treatment to postoperative SR mice significantly decreased bacterial burden (*P* < 0.05; Figure 7A).

Macrophage dysfunction in the lung is an important contributor to protract immunosuppression after primary sepsis and increased susceptibility to secondary pneumonia [32]. Cytokines IL-4 and IL-13 in lung are shown to play important roles in driving M2 polarization of alveolar macrophages (AMs) [33, 34], which could result in the susceptibility of mice to bacterial pneumonia [33]. Importantly, there was a significant decreased expression of IL-4 and IL-13 in the lung (*P* < 0.05 and *P* < 0.05, respectively; Figure 7B), along with decreased M2 macrophages markers (Arg1 and YM1) and increased M1 macrophages marker iNOS in rTFF2-treated SR mice, compared to that in PBS-treated SR mice (all *P* < 0.05; Figure 7D). The phagocytic activity of AMs in rTFF2-treated SR mice was significantly increased compared with that in PBS-treated SR mice (*P* < 0.05; Figure 7C).

**Figure 8:**
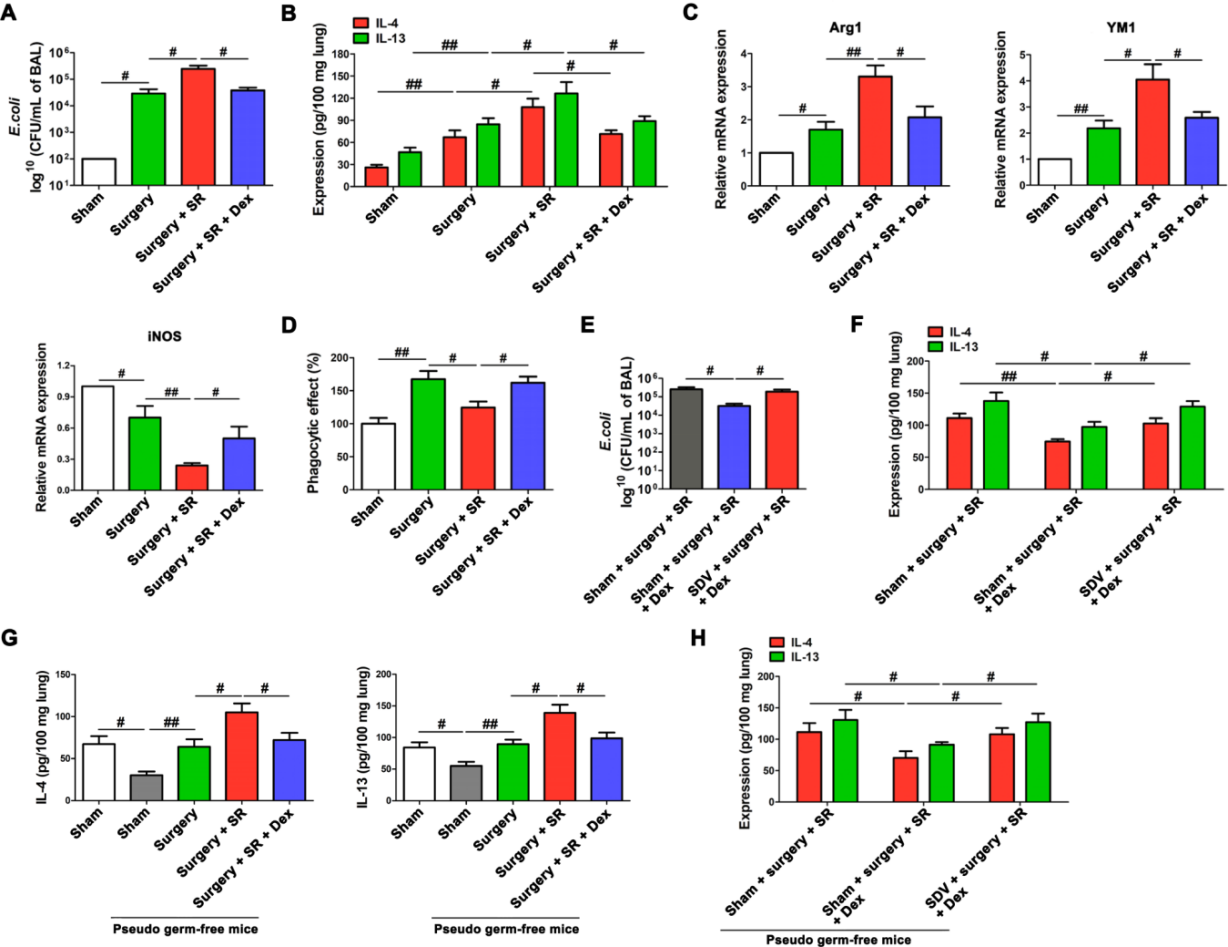
**Dexmedetomidine improved postoperative sleep-restriction (SR)-induced reduction of protective ability against Escherichia coli (*E. coli*) pneumonia.** (**A**) Enumeration of CFU per milliliter of bronchoalveolar lavage analyzed one day after *E. coli* pneumonia in postoperative SR mice with or without the treatment of dexmedetomidine (Dex). (**B**) ELISA determination of the concentrations of IL-4 and IL-13 in the lungs of postoperative SR mice with or without the treatment of Dex. (**C**) RT-PCR analysis of the mRNA expression of Arg1, YM and iNOS in alveolar macrophages from postoperative SR mice with or without the treatment of Dex. (**D**) The phagocytic activity of alveolar macrophages from postoperative SR mice with or without the treatment of Dex. (**E**) Enumeration of CFU per milliliter of bronchoalveolar lavage analyzed one day after *E. coli* pneumonia Dex-treated SR mice with or without sub-diaphragmatic vagotomy (SDV). (**F**) ELISA determination of the concentrations of IL-4 and IL-13 in the lungs of Dex-treated SR mice with or without SDV. (**G**) ELISA determination of the concentrations of IL-4 and IL-13 in the lungs of sham-operated mice and pseudo-germ-free mouse received fecal microbiota transplantation (FMT). (**H**) ELISA determination of the concentrations of IL-4 and IL-13 in the lungs of pseudo-germ-free mouse received FMT with feces of SR mice, Dex-treated SR mice or Dex-treated SR mice with SDV. All data represent mean ± SEM, n = 5; ^#^*P* < 0.05, ^##^*P* < 0.01.

Dexmedetomidine treatment during SR attenuated postoperative SR-induced increase in bacterial burden (*P* < 0.05; Figure 8A) and the expression of IL-4 and IL-13 in the lung (*P* < 0.05 and *P* < 0.05, respectively; Figure 8B). There was a significant decreased M2 macrophages markers (Arg1 and YM1) and increased M1 macrophages marker iNOS (all *P* < 0.05; Figure 8C), along with increased phagocytic activity of AMs (*P* < 0.05; Figure 8D).

After SDV, dexmedetomidine-treated SR mice showed a significant increase in bacterial burden (*P* < 0.05; Figure 8E) and a significant increase in the expression of IL-4 and IL-13 in the lung (*P* < 0.05 and *P* < 0.05, respectively; Figure 8F). Moreover, the expression of IL-4 and IL-13 in the lung were decreased in pseudo germ-free mice received FMT with feces of sham-operated mice (*P* < 0.05 and *P* < 0.05, respectively; Figure 8G), compared to that in pseudo germ-free mice without FMT. Pseudo germ-free mice received FMT with feces of dexmedetomidine-treated SR mice showed a significant decrease in the expression of IL-4 and IL-13 in the lung (*P* < 0.05 and *P* < 0.05, respectively; Figure 8G) compared with pseudo germ-free mice received FMT with feces of postoperative SR mice. Furthermore, we found that SDV abrogated dexmedetomidine treatment-mediated decrease in the expression of IL-4 and IL-13 in the lung after SR (*P* < 0.05 and *P* < 0.05, respectively; Figure 8H), indicating that gut-microbiota mediated regulation of splenic TFF2 expression via SVN is essential to dexmedetomidine-induced improvement in the antimicrobial activity in *E. coli* pneumonia.

## DISCUSSION

**Figure 9:**
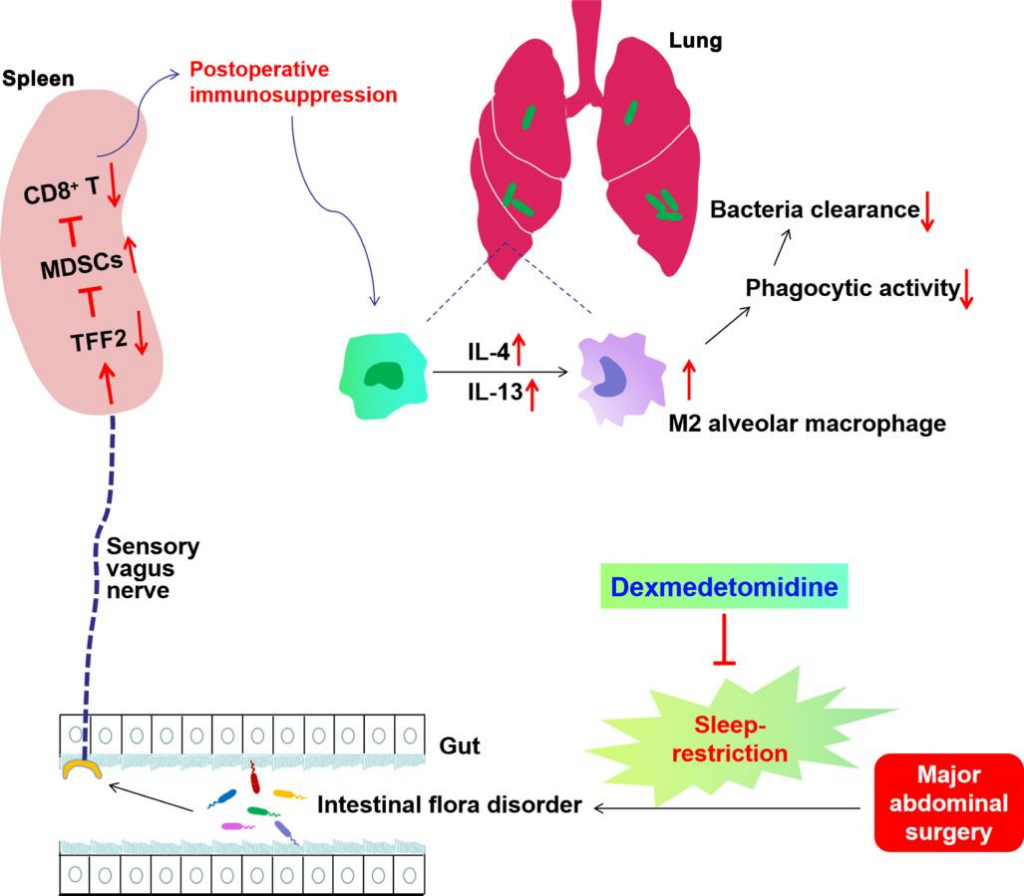
**Schematics illustrating the signaling mechanisms of sleep-restriction in postoperative immunosuppression and its treatment by dexmedetomidine.** Sleep-restriction exaggerates intestinal flora disorder, furtherly decreases splenic trefoil factor 2 (TFF2) expression, which subsequently leads to the increase in myeloid-derived suppressor cells (MDSCs) numbers and decrease in splenic CD8^+^ T cells activity. Subdiaphragmatic vagus nerve (SVN) served as an important conduit of gut microbiota-spleen communication. SR-induced exaggeration of postoperative immunosuppression was characterized by increased expression of IL-4 and IL-13 in the lung, increased M2 polarization of alveolar macrophages (AMs), decreased phagocytic activity of AMs and thus decreased antimicrobial activity in *E. coli* pneumonia. Dexmedetomidine treatment during SR alleviated SR-induced decrease in postoperative immunosuppression through gut microbiota and SVN.

Our study indicates that SR after surgery exaggerated postoperative immunosuppression through gut microbiota disturbance-mediated decrease in splenic TFF2 expression, which subsequently led to the increase in MDSCs numbers and decrease in splenic CD8^+^ T cells activity. SVN served as an important conduit of gut microbiota-spleen communication. SR-induced exaggeration of postoperative immunosuppression was characterized by furtherly decreased antimicrobial activity in *E. coli* pneumonia. Dexmedetomidine treatment during SR alleviated SR-induced decrease in postoperative immunosuppression through gut microbiota and SVN (Figure 9).

Previous studies have shown that a large proporation of patients experienced clinically significant sleep disturbance during the first postoperative week that may be due to postoperative pain and the disturbances in the circadian regulation of the sleep-wake cycle [4–8, 35]. Patients not only suffered from severely disturbed night-time sleep architecture, but also had increased rapid eye movement (REM) sleep, light sleep and reduced time awake during the daytime period after surgery [35]. REM sleep has been shown to suppress slow wave sleep and be linked to the activation of the sympathetic nervous system and HPA system [36, 37], which could inhibit innate and adaptive arm of our body's defense system [13–15]. Sleep plays important roles in immune cell activity and proliferation (especially NK-cell activity and lymphocyte proliferation), humoral immune system and immunological memory (memory T and B cells) [38]. In 42 medically and psychiatrically healthy male volunteers, a night of sleep deprivation could lead to a reduction of natural immune responses as measured by natural killer (NK) cell activity and lymphokine-activated killer (LAK) activity [16]. Sleep deprivation also severely disturbs the functional rhythm of natural regulatory T cells (nTreg) and CD4^+^CD25^-^ T cells [17]. Chronic sleep deprivation of rats could cause a breakdown of host defense indicated by a systemic bacterial invasion [39, 40]. In our present study, we found that postoperative SR for 1 week exaggerated postoperative immunosuppression, as demonstrated by the furtherly decreased phagocytic activity of AMs and antimicrobial activity against *E. coli* pneumonia.

After further investigation of the mechanism underlying SR-induced exaggeration of postoperative immunosuppression, we found that postoperative SR furtherly increased the spleen weight. One previous study indicates that the increase in the spleen weight is accompanied by an increased expansion of MDSCs in the spleen due to the decreased expression of splenic TFF2 after tumor-mediated immunosuppression [30]. The increased expansion of MDSCs in the spleen is inversely associated with splenic TFF2 status and suppress host immunity via suppression of CD8^+^ T cells in the spleen [30]. In our present study, the increase in the spleen weight after postoperative SR was also accompanied by a decrease in splenic TFF2 expression and subsequent increase in MDSCs in the spleen, as well as lower levels of IFN-γ and GrB secreted from splenic CD8^+^ T cells, which was critical in the antimicrobial activity against *E. coli* pneumonia. Our study extended our understanding of the role of splenic TFF2 inhibition-mediated MDSCs expansion in the spleen in postoperative immunosuppression, not only in tumor-mediated immunosuppression.

Previous studies have provided evidences that both chronic and acute sleep disruption could change the gut microbiota composition [18–20]. The metabolites and components of gut microbiota are not only necessary for the maintenance of immune homeostasis, they also influence the susceptibility of the host to many immune disorders through the activity of T cells, such as ischemic stroke, autoimmune kidney disease and sepsis [21, 41–43]. Gut microbiota plays an essential role in the homeostasis of the host immune system, including innate immunity and adaptive immune [23–25]. In a mice model of pneumococcal pneumonia, diverse and rich gut microbiota could protect the host against pneumococcal pneumonia, whereas gut microbiota depletion increased pneumococcal burdens in the lungs and blood and decreased the capacity of primary AMs to phagocytose *S. pneumoniae* [27]. Our present study showed that the postoperative SR induced gut microbiota dysbiosis, as reflected by a reduced gut microbiota diversity. FMT with feces from postoperative SR mice to gut microbiota-depleted mice induced more severe immunosuppression than that from surgery mice without SR, indicating an important role of gut microbiota dysbiosis in SR-induced exaggeration of postoperative immunosuppression.

SVN has been shown to be an important bridge between gut microbiota and extra-intestinal organs such as brain [28, 29]. SVN also exerts key roles in promoting splenic TFF2 expression in tumor-mediated immunosuppression [30]. Vagus nerve stimulation increased TFF2 expression in the spleen, whereas bilateral subdiaphragmatic vagotomy abrogated splenic *Tff2* response to dextran sodium sulfate [30]. In our present study, we found that SVN played essential roles in increasing the expression of TFF2 in the spleen after surgery, suggesting that SVN also mediated the communication of gut microbiota with spleen. This finding furtherly expands our understanding of the role of SVN serving as a bridge between gut microbiota and extra-intestinal organs including spleen (not just brain).

Dexmedetomidine, a highly selective α 2 adrenoceptor agonist, has beneficial pharmacological properties, providing sedative, anxiolytic, sympatholytic and analgesic-sparing effects without relevant respiratory depression [44]. Sedation with dexmedetomidine mimics the natural sleep state, showing an electroencephalography signal pattern similar to that during stage N2 non-rapid eye movement (NREM) sleep. It is shown that dexmedetomidine could promote NREM stage N3 sleep in a dose dependent manner without effects on psychomotor performance [31]. In nonmechanically ventilated elderly patients admitted to the ICU after surgery, low-dose dexmedetomidine infusion (0.1 mg/kg/h) during the night after surgery could prolong total sleep time, increase N2 sleep and improve subjective sleep quality [45]**.** Dexmedetomidine is shown to possess anti-inflammatory and immunomodulatory effects, and could improve outcomes in the setting of infection [46]. In our present study, we showed that dexmedetomidine treatment during SR alleviated SR-induced exaggeration of postoperative immunosuppression through improving gut microbiota, which then increased splenic TFF2 expression, decreased MDSCs expansion in the spleen, and increased the activity of splenic CD8^+^ T cells and phagocytic activity of AMs via the SVN. However, whether dexmedetomidine treatment during SR exerted beneficial effects on postoperative immunosuppression through improving the sleep quality needs further study.

In summary, our results suggest that SR after surgery exaggerated postoperative immunosuppression through the inhibition of splenic TFF2 expression thus increasing MDSCs in the spleen and reducing splenic CD8^+^ T cells activity, which was mediated by gut microbiota. Dexmedetomidine could be a good drug for the alleviation of postoperative SR-mediated exaggeration of immunosuppression through improving gut microbiota and subsequently increasing splenic TFF2 expression via SVN.

## MATERIALS AND METHODS

### Animals

Male C57BL/6 mice, 78 weeks old, were purchased from Vital River Laboratory Animal Technology Co Ltd., Beijing, China. Mice were housed in the specific pathogen-free conditions with *ad libitum* access to food and water. The room temperature was maintained at 23 °C under a 12-hour light-dark cycle. All procedures were performed in accordance with the National Institute of Health (NIH) Guide for the Care and Use of Laboratory Animals (publications no. 80-23) revised 1996 and approved by the Animal Care and Use Committee of Henan Provincial People's Hospital.

### Partial hepatectomy

Two-thirds partial hepatectomy was performed as previously described [47]. Briefly, a middle abdominal skin and muscle incision (about 3 cm long) was made, and the liver was exposed. Left and anterior (median) lobes were resected by proximal ligation. Then, the peritoneum was closed with a 5-0 suture and the skin was closed by a 4-0 suture. Laparotomy with gentle liver manipulation was performed as a sham operation.

### Sleep-restriction

Using the multiple platform method, SR lasted for 7 days starting immediately after surgery, with only 4 h of sleep (during the last 4 h of the light phase) in every 24 h. Mice were placed in a polypropylene cage (500×300×170 mm; 4 mice/cage) containing 9 circular platforms (diameter: 3 cm, height: 2.5 cm). Water filled the cage 1 cm below the upper surface of the platforms, allowing the mice to move from one platform to another by jumping and access food and water during SR. When mice reached the rapid eye movement stage of sleep, they fell into the water due to muscle atonia. The mice were awoken and would try to climb up the platform. Throughout the experiments, the water was replaced with clean water every day.

### Dexmedetomidine and rTFF2 treatment

Dexmedetomidine (4 μg/mL diluted in NS; 50 μg/kg; Xinchen Pharmaceutical Company, Jiangsu, China) or normal saline was intravenously injected at the beginning of 4 h of sleep during the SR protocols.

After 7 days of SR, recombinant human TFF2 protein (rTFF2; 0.2 mg/kg; R&D Systems, Minneapolis, MN, USA) was injected intravenously via the tail vein.

### Antibiotic treatment

The pseudo-germ-free mouse model was established as previously described [48]. Briefly, mice were treated with broad-spectrum antibiotics in drinking water for 2 weeks (ampicillin 1 g/L, neomycin sulfate 1 g/L, metronidazole 1 g/L; Sigma-Aldrich, St Louis, MO, USA) *ad libitum* to achieve pseudo-germ-free status. Antibiotics were renewed every 2 days.

### Fecal microbiota transplantation (FMT)

A clean cage containing sterilized filter paper was used to collect feces. Feces of each group were collected immediately after defecation and combined into one sample in a sterile microtube. The Fecal samples were frozen immediately in liquid nitrogen and then stored at −80°C until analysis and transplantation. 1 g Fecal samples were homogenised in 10 mL sterile normal saline under anaerobic conditions, and the fecal material were then suspended by vortexing. 0.2 mL of the suspension was introduced by gavage into each mouse recipient for 14 consecutive days.

### 16S ribosomal RNA (16S rRNA) gene sequencing

DNA extraction was performed using TIANamp stool DNA kits (Tiangen Biotechnology Company, Beijing, China). For the Illumina MiSeq sequencing, the PCR amplification of the V3-V4 region of the bacterial 16S rRNA was performed using the following primers: 338F (5′-ACTCCTACGGGAGGCAGC-3′) and 806R (5′-GG ACTACHVGGGTWTCTAAT-3′). Sequencing was conducted using a paired-end 2 × 300 base pairs (bp) cycle run on an Illumina MiSeq platform (Berry Genomics Co., Ltd, Beijing, China). Reads obtained from the samples were sorted by unique barcodes of each PCR product. The barcode, linker, and PCR primer sequences were removed from the the original sequencing reads. The resultant sequences were screened for quality, and only sequences with ≥70 bp were selected for bioinformatics analysis. NCBI BLAST and SILVA databases were used to classify all sequences. Distance calculation, operational taxonomic units cluster, rarefaction analysis and α-diversity were performed by the MOTHUR program.

### Vagus nerve stimulation

After 7 days of SR, VNS was performed as previously described [30]. Briefly, mice were anesthetized intraperitoneally (i.p.) with ketamine (100 mg/kg) and xylazine (8 mg/kg). A midline abdominal incision was made and the ventral branch of SVN was isolated. The nerve was stimulated for 4 h with electrical pulses of 2 ms duration at 5 Hz by a stimulation module (STM100A) controlled by the AcqKnowledge software (Biopac Systems Inc., Goleta, California, USA). The electrical voltage was 1V. The abdomen is closed in two layers. In Sham-operated mice only an abdominal incision was made and the ventral branch of SVN isolated. Spleen was harvested 3 h later.

### Bilateral sub-diaphragmatic vagotomy (SDV)

Bilateral sub-diaphragmatic vagotomy was performed. Briefly, following adequate depth of anesthesia, a right abdominal transverse incision (~1 cm) was made about 0.5 cm below the xiphisternum starting from the *linea alba*. The liver was retracted with a saline dampened cotton swab and the esophagus was exposed while carefully keeping costal arc and liver out of sight. The ventral branch of the vagal nerve was exposed and about 3 mm were resected with the aid of a surgical microscope (RWD Life Science Co., Ltd, Shenzhen, China). The dorsal branch of the vagal nerve was exposed beneath the esophagus, and then was isolated (3~5 mm) and resected. Successful SDV was confirmed by an increase in stomach size, and impairment of anorexigenic effect of cholecystokinin (4 μg/kg, i.p.). For sham-operated mice, vagus nerve was gently exposed without further manipulation.

### Enzyme-linked immunospot (ELISPOT) assay

Splenic CD8^+^ T cells were labelled with PE-Cy7-conjugated CD8 antibodies and sorted using FASCAria flow cytometer (Becton Dickinson, San Jose, CA, USA). Sorted spleen CD8^+^ T cells were stimulated with anti-CD3/CD28 antibodies (Biolegend, San Diego, CA, USA; 1 μg/mL) for 48 h. ELISPOT assays were performed with Mouse IFN-γ/GrB dual-color Elispot kit (R&D Systems, Minneapolis, MN, USA) according to manufacturer’s protocol.

### Western blotting

Spleens were homogenized in cell lysis buffer with added protease inhibitors (KeyGen Biotech, Nanjing, China). Proteins (50 μg) were separated by electrophoresis on 10% SDS-PAGE gels (Beyotime Institute of Biotechnology, Shanghai, China), transblotted into PVDF membranes (Millipore; Merck KGaA), and incubated overnight at 4 °C with primary antibodies against TFF2 (1:500; Wuhan Boster Biological Technology, Ltd.) and β-actin (1:2,000; Santa Cruz Biotechnology, Inc., Dallas, TX, USA). After primary antibody incubation, membranes were washed with TBST and incubated with peroxidase-conjugated secondary anti-rabbit antibody (1:3000; Proteintech group, Wuhan, China) for 2 h at room temperature. After three washes in TBST, bands were detected using enhanced chemiluminescence (ECL) and bands were captured using an UVP gel documentation system (UVP, LLC, Phoenix, AZ, USA). The relative densities of bands were analyzed with Image J software (version 1.41; National Institutes of Health, Bethesda, MD, USA).

### Flow cytometry

At 7 days after surgery, the spleens were dissected and washed in cold phosphate buffered saline (PBS, pH 7.4). Spleens were cut into small pieces and digested using 15 ml digestion buffer (5% FBS (Sigma, St. Louis, MO, USA) + collagenase IV (1.75 mg/ml; Roche, Indianapolis, IN, USA) + DNase I (0.5 mg/ml; Sigma, St. Louis, MO, USA)). After 40 min incubation at 37 °C the remaining pieces were filtered through a 200-mesh stain steel sieve. The supernatants were washed once in cold flow cytometry (FACS) buffer and separated by Percoll gradient (Sigma, St. Louis, MO, USA). Lymphocytes were harvested from the interphase of the Percoll gradient and resuspended in FACS buffer. Obtained cells were stained with optimal concentration of anti-Gr-1-PerCP 5.5 (eBioscience, San Diego, CA) and anti-CD11b-APC (eBioscience, San Diego, CA) using permeabilization buffer (eBioscience, San Diego, CA). Stained cells were run on a FACSverse flow cytometer (BD Biosciences). Data were analysed with FloJo X analysis software (FreeStar Ashland, OR, USA).

### RT-PCR

Total RNA was extracted by using Trizol reagent according to the manufacturer’s protocol (Invitrogen, Carlsbad, CA). cDNA was synthesized from total RNA with Taqman reverse transcriptase (Applied Biosystems, Foster City, CA). TFF2, Arg1, YM1, iNOS and β-actin cDNA were amplified using Power SYBR Green (Applied Biosystems, Foster City, CA). The cycling conditions used were 50 °C for 2 minutes and 95 °C for 10 minutes, followed by 40 cycles at 95 °C for 15 seconds and 60 °C for 1 minute with specific primers for TFF2, Arg1, YM1, iNOS and β-actin (primer sequences listed in Table 1). Reactions were run and analysed on ABI StepOne™ Real-Time PCR System (Applied Biosystems, Foster City, CA, USA). The β-actin mRNA was used as the internal control. Gene expression was determined by 2^-ΔΔCt^ methodology, normalized against the β-actin.

**Table 1:** The sequences of primers for real-time PCR.

**Target genes**	**Sense primers**	**Antisense primers**
TFF2	5′-GTCAGCTCGCAAGAATTGTG-3′	5′-GGCAGTAGCAACTCTCAGTA-3′
Arg1	5′-ATGGAAGAGACCTTCAGCTAC-3′	5′-GCTGTCTTCCCAAGAGTTGGG-3′
YM1	5′-CATGAGCAAGACTTGCGTGAC-3′	5′-GGTCCAAACTTCCATCCTCCA-3′
iNOS	5′-CCGAAGCAAACATCACATTCA-3′	5′-GGTCTAAAGGCTCCGGGCT-3′
β-actin	5′-TGTCCACCTTCCAGCAGATGT-3’	5′-AGCTCAGTAACAGTCCGCCTADA-3′

### Enzyme-linked immunosorbent assay (ELISA)

One day after *E. coli* pneumonia, lung tissue (200 mg) was homogenized in 500 μL PBS and subsequently centrifuged at 10,000g at 4 °C for 15 minutes. The concentration of IL-4 and IL-13 in the supernatant were determined by the ELISA kit according to the manufacturer’s protocol (R&D Systems, Minneapolis, MN, USA).

### Induction of pneumonia and BALF bacterial load measurement

After 7 days of SR, a non-lethal acute pneumonia was induced. Escherichia coli (*E.coli*) DH5-alpha, grown for 18 h in luria broth medium at 37 °C, was washed twice (1.000 g, 10 min, 37°C), diluted in sterile isotonic saline and calibrated by nephelometry. Anesthetized mice were injured with intratracheal injection of E.coli (75 mL, OD600 = 0.6-0.7) [32].

One day after *E. coli* pneumonia, BAL was serial diluted with sterile PBS under sterile condition and was then cultured on blood-agar base plates overnight at 37 °C. Colony-forming units (CFUs) per milliliter of bronchoalveolar lavage were analysed.

### AMs phagocytosis assay

One day after *E. coli* pneumonia, bronchoalveolar lavage was performed via a tracheal cannula with 0.5 mL PBS containing 1mM EDTA for 3 times, and a total volume of 1.2 mL BALF was collected. The collected BALF was centrifuged at 500g for 5 min at room temperature and the supernatant is removed. After washing twice with complete Roswell Park Memorial Institute (RPMI)-1640 medium supplemented with 1% (V/V) penicillin-streptomycin (Solarbio, Beijing, China) and 10% (V/V) fetal bovine serum (FBS, HyClone, Logan, Utah), cells were seeded in 12-well plates (1~5×10^5^) and allowed to adhere for 90 min inside a 5% CO_2_ incubator. Non-adherent cells were removed by washing two times with PBS and the adherent macrophages phagocytic activity of *E. coli* was performed with commercial Vybrant phagocytosis assay kit (Molecular Probes, Darmstadt, Germany) according to the manufacturer’s instructions. FITC-conjugated *E. coli* was resuspended in Hank's Balanced Salt Solution (HBSS; GIBCO) and sonicated until all the fluorescent particles are homogeneously dispersed. After removing the RPMI-1640 solutions, all the microplate wells except the control wells were added with 100 μL fluorescent bioparticle suspension. Trypan blue was added for the evaluation by the fluorescence plate reader (excitation wavelength at 485 nm, emission at 520 nm). The percentage of phagocytosis was calculated as follows: % Effect = (experimental reading—negative control reading) /(positive control reading—negative control reading)×100%. Negative control wells were prepared with no cell-containing and no fluorescent bioparticle exposed, whereas positive control wells were seeded with cells but not exposed to fluorescent bioparticle.

### Statistical analysis

All data were presented as mean ± SEM. One-way analysis of variance (ANOVA) followed by a Newman-Keuls multiple comparison test was used when comparing more than two groups. Student's t-test was used to compare differences between two groups. *P* values <0.05 were considered significant. For the statistical analysis, IBM SPSS Statistics version 20 (SPSS Inc., 2003, Chicago, USA) was used.

## References

[1] Hogan BV, Peter MB, Shenoy HG, Horgan K, Hughes TA. Surgery induced immunosuppression. Surgeon. 2011; 9:38–43. 10.1016/j.surge.2010.07.011 21195330

[2] Chopra SS, Haacke N, Meisel C, Unterwalder N, Fikatas P, Schmidt SC. Postoperative immunosuppression after open and laparoscopic liver resection: assessment of cellular immune function and monocytic HLA-DR expression. JSLS. 2013; 17:615–21. 10.4293/108680813X13693422519677 24398205 PMC3866067

[3] Shakhar G, Ben-Eliyahu S. Potential prophylactic measures against postoperative immunosuppression: could they reduce recurrence rates in oncological patients? Ann Surg Oncol. 2003; 10:972–92. 10.1245/ASO.2003.02.007 14527919

[4] Park MJ, Yoo JH, Cho BW, Kim KT, Jeong WC, Ha M. Noise in hospital rooms and sleep disturbance in hospitalized medical patients. Environ Health Toxicol. 2014; 29:e2014006. 10.5620/eht.2014.29.e2014006 25163680 PMC4152942

[5] Fielden JM, Gander PH, Horne JG, Lewer BM, Green RM, Devane PA. An assessment of sleep disturbance in patients before and after total hip arthroplasty. J Arthroplasty. 2003; 18:371–76. 10.1054/arth.2003.50056 12728433

[6] Kain ZN, Caldwell-Andrews AA. Sleeping characteristics of adults undergoing outpatient elective surgery: a cohort study. J Clin Anesth. 2003; 15:505–09. 10.1016/j.jclinane.2003.02.002 14698361

[7] Cronin AJ, Keifer JC, Davies MF, King TS, Bixler EO. Postoperative sleep disturbance: influences of opioids and pain in humans. Sleep. 2001; 24:39–44. 10.1093/sleep/24.1.39 11204052

[8] Knill RL, Moote CA, Skinner MI, Rose EA. Anesthesia with abdominal surgery leads to intense REM sleep during the first postoperative week. Anesthesiology. 1990; 73:52–61. 10.1097/00000542-199007000-00009 2360740

[9] Besedovsky L, Lange T, Born J. Sleep and immune function. Pflugers Arch. 2012; 463:121–37. 10.1007/s00424-011-1044-0 22071480 PMC3256323

[10] Friese RS. Sleep and recovery from critical illness and injury: a review of theory, current practice, and future directions. Crit Care Med. 2008; 36:697–705. 10.1097/CCM.0B013E3181643F29 18176314

[11] Leproult R, Copinschi G, Buxton O, Van Cauter E. Sleep loss results in an elevation of cortisol levels the next evening. Sleep. 1997; 20:865–70. 10.1093/sleep/20.10.865 9415946

[12] Dettoni JL, Consolim-Colombo FM, Drager LF, Rubira MC, Souza SB, Irigoyen MC, Mostarda C, Borile S, Krieger EM, Moreno H Jr, Lorenzi-Filho G. Cardiovascular effects of partial sleep deprivation in healthy volunteers. J Appl Physiol (1985). 2012; 113:232–6. 10.1152/japplphysiol.01604.2011 22539169

[13] Taub DD. Neuroendocrine interactions in the immune system. Cell Immunol. 2008; 252:1–6. 10.1016/j.cellimm.2008.05.006 18619587 PMC2562609

[14] Bellinger DL, Millar BA, Perez S, Carter J, Wood C, ThyagaRajan S, Molinaro C, Lubahn C, Lorton D. Sympathetic modulation of immunity: relevance to disease. Cell Immunol. 2008; 252:27–56. 10.1016/j.cellimm.2007.09.005 18308299 PMC3551630

[15] Bartal I, Melamed R, Greenfeld K, Atzil S, Glasner A, Domankevich V, Naor R, Beilin B, Yardeni IZ, Ben-Eliyahu S. Immune perturbations in patients along the perioperative period: alterations in cell surface markers and leukocyte subtypes before and after surgery. Brain Behav Immun. 2010; 24:376–86. 10.1016/j.bbi.2009.02.010 19254757

[16] Irwin M, McClintick J, Costlow C, Fortner M, White J, Gillin JC. Partial night sleep deprivation reduces natural killer and cellular immune responses in humans. FASEB J. 1996; 10:643–53. 10.1096/fasebj.10.5.8621064 8621064

[17] Bollinger T, Bollinger A, Skrum L, Dimitrov S, Lange T, Solbach W. Sleep-dependent activity of T cells and regulatory T cells. Clin Exp Immunol. 2009; 155:231–38. 10.1111/j.1365-2249.2008.03822.x 19040608 PMC2675254

[18] El Aidy S, Bolsius YG, Raven F, Havekes R. A brief period of sleep deprivation leads to subtle changes in mouse gut microbiota. J Sleep Res. 2019. [Epub ahead of print]. 10.1111/jsr.12920 31515894 PMC7757181

[19] Ma W, Song J, Wang H, Shi F, Zhou N, Jiang J, Xu Y, Zhang L, Yang L, Zhou M. Chronic paradoxical sleep deprivation-induced depression-like behavior, energy metabolism and microbial changes in rats. Life Sci. 2019; 225:88–97. 10.1016/j.lfs.2019.04.006 30953642

[20] Poroyko VA, Carreras A, Khalyfa A, Khalyfa AA, Leone V, Peris E, Almendros I, Gileles-Hillel A, Qiao Z, Hubert N, Farré R, Chang EB, Gozal D. Chronic Sleep Disruption Alters Gut Microbiota, Induces Systemic and Adipose Tissue Inflammation and Insulin Resistance in Mice. Sci Rep. 2016; 6:35405. 10.1038/srep35405 27739530 PMC5064361

[21] Rooks MG, Garrett WS. Gut microbiota, metabolites and host immunity. Nat Rev Immunol. 2016; 16:341–52. 10.1038/nri.2016.42 27231050 PMC5541232

[22] Salva S, Alvarez S. The Role of Microbiota and Immunobiotics in Granulopoiesis of Immunocompromised Hosts. Front Immunol. 2017; 8:507. 10.3389/fimmu.2017.00507 28533775 PMC5421150

[23] Thaiss CA, Zmora N, Levy M, Elinav E. The microbiome and innate immunity. Nature. 2016; 535:65–74. 10.1038/nature18847 27383981

[24] Zhao Q, Elson CO. Adaptive immune education by gut microbiota antigens. Immunology. 2018; 154:28–37. 10.1111/imm.12896 29338074 PMC5904715

[25] Honda K, Littman DR. The microbiota in adaptive immune homeostasis and disease. Nature. 2016; 535:75–84. 10.1038/nature18848 27383982

[26] McAleer JP, Kolls JK. Contributions of the intestinal microbiome in lung immunity. Eur J Immunol. 2018; 48:39–49. 10.1002/eji.201646721 28776643 PMC5762407

[27] Schuijt TJ, Lankelma JM, Scicluna BP, de Sousa e Melo F, Roelofs JJ, de Boer JD, Hoogendijk AJ, de Beer R, de Vos A, Belzer C, de Vos WM, van der Poll T, Wiersinga WJ. The gut microbiota plays a protective role in the host defence against pneumococcal pneumonia. Gut. 2016; 65:575–83. 10.1136/gutjnl-2015-309728 26511795 PMC4819612

[28] Zhou L, Foster JA. Psychobiotics and the gut-brain axis: in the pursuit of happiness. Neuropsychiatr Dis Treat. 2015; 11:715–23. 10.2147/NDT.S61997 25834446 PMC4370913

[29] Bonaz B, Bazin T, Pellissier S. The Vagus Nerve at the Interface of the Microbiota-Gut-Brain Axis. Front Neurosci. 2018; 12:49. 10.3389/fnins.2018.00049 29467611 PMC5808284

[30] Dubeykovskaya Z, Si Y, Chen X, Worthley DL, Renz BW, Urbanska AM, Hayakawa Y, Xu T, Westphalen CB, Dubeykovskiy A, Chen D, Friedman RA, Asfaha S, et al. Neural innervation stimulates splenic TFF2 to arrest myeloid cell expansion and cancer. Nat Commun. 2016; 7:10517. 10.1038/ncomms10517 26841680 PMC4742920

[31] Akeju O, Hobbs LE, Gao L, Burns SM, Pavone KJ, Plummer GS, Walsh EC, Houle TT, Kim SE, Bianchi MT, Ellenbogen JM, Brown EN. Dexmedetomidine promotes biomimetic non-rapid eye movement stage 3 sleep in humans: A pilot study. Clin Neurophysiol. 2018; 129:69–78. 10.1016/j.clinph.2017.10.005 29154132 PMC5743618

[32] Roquilly A, McWilliam HE, Jacqueline C, Tian Z, Cinotti R, Rimbert M, Wakim L, Caminschi I, Lahoud MH, Belz GT, Kallies A, Mintern JD, Asehnoune K, Villadangos JA. Local Modulation of Antigen-Presenting Cell Development after Resolution of Pneumonia Induces Long-Term Susceptibility to Secondary Infections. Immunity. 2017; 47:135–147.e5. 10.1016/j.immuni.2017.06.021 28723546

[33] Nascimento DC, Melo PH, Piñeros AR, Ferreira RG, Colón DF, Donate PB, Castanheira FV, Gozzi A, Czaikoski PG, Niedbala W, Borges MC, Zamboni DS, Liew FY, et al. IL-33 contributes to sepsis-induced long-term immunosuppression by expanding the regulatory T cell population. Nat Commun. 2017; 8:14919. 10.1038/ncomms14919 28374774 PMC5382289

[34] Biswas SK, Mantovani A. Macrophage plasticity and interaction with lymphocyte subsets: cancer as a paradigm. Nat Immunol. 2010; 11:889–96. 10.1038/ni.1937 20856220

[35] Gögenur I, Wildschiøtz G, Rosenberg J. Circadian distribution of sleep phases after major abdominal surgery. Br J Anaesth. 2008; 100:45–49. 10.1093/bja/aem340 18037670

[36] Vgontzas AN, Bixler EO, Papanicolaou DA, Kales A, Stratakis CA, Vela-Bueno A, Gold PW, Chrousos GP. Rapid eye movement sleep correlates with the overall activities of the hypothalamic-pituitary-adrenal axis and sympathetic system in healthy humans. J Clin Endocrinol Metab. 1997; 82:3278–80. 10.1210/jcem.82.10.4307 9329353

[37] Van Cauter E, Spiegel K, Tasali E, Leproult R. Metabolic consequences of sleep and sleep loss. Sleep Med. 2008 (Suppl 1); 9:S23–28. 10.1016/S1389-9457(08)70013-3 18929315 PMC4444051

[38] Besedovsky L, Lange T, Haack M. The Sleep-Immune Crosstalk in Health and Disease. Physiol Rev. 2019; 99:1325–80. 10.1152/physrev.00010.2018 30920354 PMC6689741

[39] Everson CA. Sustained sleep deprivation impairs host defense. Am J Physiol. 1993; 265:R1148–54. 10.1152/ajpregu.1993.265.5.R1148 8238617

[40] Everson CA, Toth LA. Systemic bacterial invasion induced by sleep deprivation. Am J Physiol Regul Integr Comp Physiol. 2000; 278:R905–16. 10.1152/ajpregu.2000.278.4.R905 10749778

[41] Krebs CF, Paust HJ, Krohn S, Koyro T, Brix SR, Riedel JH, Bartsch P, Wiech T, Meyer-Schwesinger C, Huang J, Fischer N, Busch P, Mittrücker HW, et al. Autoimmune Renal Disease Is Exacerbated by S1P-Receptor-1-Dependent Intestinal Th17 Cell Migration to the Kidney. Immunity. 2016; 45:1078–92. 10.1016/j.immuni.2016.10.020 27851911 PMC6381450

[42] Benakis C, Brea D, Caballero S, Faraco G, Moore J, Murphy M, Sita G, Racchumi G, Ling L, Pamer EG, Iadecola C, Anrather J. Commensal microbiota affects ischemic stroke outcome by regulating intestinal γδ T cells. Nat Med. 2016; 22:516–23. 10.1038/nm.4068 27019327 PMC4860105

[43] Haak BW, Wiersinga WJ. The role of the gut microbiota in sepsis. Lancet Gastroenterol Hepatol. 2017; 2:135–43. 10.1016/S2468-1253(16)30119-4 28403983

[44] Weerink MA, Struys MM, Hannivoort LN, Barends CR, Absalom AR, Colin P. Clinical Pharmacokinetics and Pharmacodynamics of Dexmedetomidine. Clin Pharmacokinet. 2017; 56:893–913. 10.1007/s40262-017-0507-7 28105598 PMC5511603

[45] Wu XH, Cui F, Zhang C, Meng ZT, Wang DX, Ma J, Wang GF, Zhu SN, Ma D. Low-dose Dexmedetomidine Improves Sleep Quality Pattern in Elderly Patients after Noncardiac Surgery in the Intensive Care Unit: A Pilot Randomized Controlled Trial. Anesthesiology. 2016; 125:979–91. 10.1097/ALN.0000000000001325 27571256

[46] Sanders RD, Hussell T, Maze M. Sedation and immunomodulation. Crit Care Clin. 2009; 25:551–70, ix. 10.1016/j.ccc.2009.05.001 19576530

[47] Mitchell C, Willenbring H. A reproducible and well-tolerated method for 2/3 partial hepatectomy in mice. Nat Protoc. 2008; 3:1167–70. 10.1038/nprot.2008.80 18600221

[48] Yu F, Han W, Zhan G, Li S, Xiang S, Zhu B, Jiang X, Yang L, Luo A, Hua F, Yang C. Abnormal gut microbiota composition contributes to cognitive dysfunction in streptozotocin-induced diabetic mice. Aging (Albany NY). 2019; 11:3262–79. 10.18632/aging.101978 31123221 PMC6555457

